# Boolean Modelling Reveals New Regulatory Connections between Transcription Factors Orchestrating the Development of the Ventral Spinal Cord

**DOI:** 10.1371/journal.pone.0111430

**Published:** 2014-11-14

**Authors:** Anna Lovrics, Yu Gao, Bianka Juhász, István Bock, Helen M. Byrne, András Dinnyés, Krisztián A. Kovács

**Affiliations:** 1 Biotalentum Ltd., Gödöllö, Hungary; 2 Molecular Animal Biotechnology Laboratory, Szent Istvan University, Gödöllö, Hungary; 3 Oxford Centre for Collaborative Applied Mathematics, Mathematical Institute, University of Oxford, Oxford, United Kingdom; 4 Department of Farm Animal Health, Faculty of Veterinary Medicine, Utrecht University, Utrecht, The Netherlands; 5 Institute of Science and Technology, Klosterneuburg, Austria; Virginia Tech Carilion Research Institute, United States of America

## Abstract

We have assembled a network of cell-fate determining transcription factors that play a key role in the specification of the ventral neuronal subtypes of the spinal cord on the basis of published transcriptional interactions. Asynchronous Boolean modelling of the network was used to compare simulation results with reported experimental observations. Such comparison highlighted the need to include additional regulatory connections in order to obtain the fixed point attractors of the model associated with the five known progenitor cell types located in the ventral spinal cord. The revised gene regulatory network reproduced previously observed cell state switches between progenitor cells observed in knock-out animal models or in experiments where the transcription factors were overexpressed. Furthermore the network predicted the inhibition of Irx3 by Nkx2.2 and this prediction was tested experimentally. Our results provide evidence for the existence of an as yet undescribed inhibitory connection which could potentially have significance beyond the ventral spinal cord. The work presented in this paper demonstrates the strength of Boolean modelling for identifying gene regulatory networks.

## Introduction

**Figure 1:**
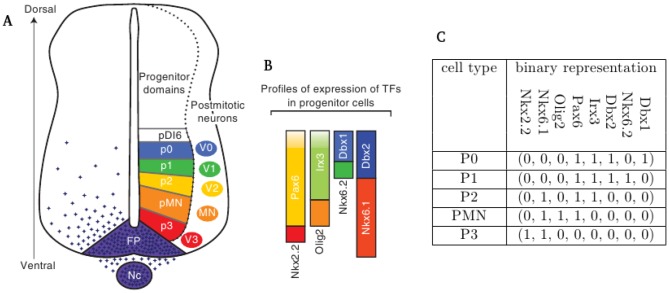
Transcription factor profiles in the ventral spinal cord. (A) Neural progenitor cells along the dorsal-ventral axis of the ventral spinal cord and (B) the corresponding TFs. Figure 2A,B from [3] modified, with permission. (C) Binary representation of TF expression (present or absent) in each progenitor cell type in the order (Nkx2.2,Nkx6.1,Olig2,Pax6,Irx3,Dbx2,Nkx6.2,Dbx1).

One anatomic location in vertebrate organisms where the classical concept of gradient-mediated morphogenesis is very clearly manifested is the neural tube, the precursor of the central nervous system, generated by the invagination of the dorsal ectoderm. The rostral part (*i.e.* the portion that is situated toward the head of the organism) swells into structures to develop eventually into distinct brain structures. The caudal part (*i.e.* the portion that is situated toward the tail of the organism) of the neural tube gives rise to the spinal cord, and at all stages of the development, shows a more uniform structure along the anteroposterior axis than the rostral part. The most spectacular and most studied patterning axis of the presumptive spinal cord is dorsoventral, mediated by secreted factors, such as sonic hedgehog (Shh), wingless (Wnt) [1], bone morphogenetic proteins (BMP) and activin [2]. The most dorsally located portion (termed roof plate) is source of BMPs, Wnt and activin while Shh is produced by the most ventrally located portion (termed floor plate) and also by the notochord, located ventrally from the spinal cord (figure 1).

Importantly, the secreted factors not only define ventral and dorsal identity, but also, via their concentration gradient, direct the differentiation of distinct dorsal and ventral neuronal subtypes. Accordingly, Shh concentration defines motoneurons and a set of ventral interneurons with lower concentrations responsible for less ventral phenotypes [4], while roof plate derived secreted factors specify the identity of dorsal interneurons with higher concentrations responsible for more dorsal cells [5]. Notably, Shh induces glioma-associated activator (Gli) proteins [6] while Wnt induces the glioma-associated repressor (Gli3) molecule [1] therefore these two morphogen gradients impact the same, or at least an overlapping, set of target genes. Evidently, there can be further undiscovered downstream factors of the two soluble signalling molecules.

The activity of Gli activator and repressor proteins, showing a graded pattern in the spinal cord along the dorsoventral axis, is translated into a layer-specific code of cell fate determining transcription factors (TFs), mostly homeobox proteins [7]. For instance the layer of motoneurons expresses Olig2, but not other factors present in the adjacent layers (Irx3 dorsally and Nkx2.2 ventrally as shown in figure 1). In agreement with this concept, binding sites for Gli proteins have been identified in many of the promoters driving the expression of layer specific TFs [6], [8] and it has been proposed that the activity of Gli proteins and cross-repressing TFs together determine cell type identity [9]. Importantly, once positional information has been read out by the cells, their fate and the corresponding transcriptional changes can be followed in time even in explants prepared from the appropriate region of the spinal cord [10]. To some extent, the layer specific events can be recapitulated during the directed neural differentiation of embryonic stem cells, furthermore, the same cell fate determining TFs (such as Olig2) are expressed in a temporally defined manner in these cultures [11]. Therefore hope arises that what has been learned and what is yet to be understood from the patterning of the ventral spinal cord, might also be used for guided differentiation of desired neuronal subtypes from pluripotent cells.

As reflected by the signalling events described above, specification of the ventral neuronal subtypes (hereafter referred to as *ventralization*) necessitates the orchestrated action of a complex transcriptional gene regulatory network (GRN). Due to the complex nature of the GRN, a formal network model is needed to understand its dynamics. Mathematical modelling of GRNs have been widely used to characterise complex networks, predict missing links and simulate dynamic changes of TF expression levels as reviewed in [12].

Mathematical models for the core GRN of ventralization downstream to Shh signalling have been developed in previous studies. Lai et al. [13] studied a GRN model of Shh, the elements of the machinery that transduces its signal into the cells, and its primary targets, Gli activator and repressor proteins. The ordinary differential equation model has the ability to switch between two distinct states as a function of Shh signalling. Furthermore, this core model has been extended to incorporate the transport of Shh within the neural tissue [14]. Based on a similar GRN, a hierarchical genetic computation algorithm was employed [15] to reproduce changes in the levels of TFs as a response to Shh signalling, in particular, experimentally observed desensitization [16]. However, while remarkable conclusions have been drawn from these models such as bistability and how Shh gradient formation determines the cell response, they do not account for the development of multiple different cell types in the ventral neural tube. Balaskas et al. [17] formulated a model of three cell-fate determining TFs (Pax6, Olig2, Nkx2.2) which could reproduce attaining three different cells fates based on the concentration and temporal profile of Shh. The topology of a corresponding simplified mathematical model was studied in more detail [18], where possible extensions of the GRN was also addressed. Still, regulatory links between the actual TFs responsible for layer specification of five progenitor domains within cells located dorsally to the motoneuron area have not been modelled.

In Boolean networks (proposed by Kauffman [19] to study GRNs) TFs are represented by nodes and the activating and inhibiting connections between them by edges. The simplest Boolean models assume *i*) two discrete states for each node of the network, *ii*) instead of using kinetic parameters, nodes are updated synchronously and *iii*) effects of the TFs are taken into account as logic rules. As a result of this, Boolean modelling is a good approach to simulate regulatory networks with on/off dynamics and unknown kinetic parameters and can be used to infer the structure of a GRN. Additionally, it is straightforward to study the effect of knockout or overexpression of a gene [20]. Fixed point attractors of a Boolean model may represent cellular states or phenotypes and limit cycles can correspond to oscillatory processes, such as the cell cycle [21].

The simplest Boolean networks discussed above may be extended to account for more realistic scenarios ranging from the straightforward option of using more than two discrete states to include stochasticity in gene regulation [22]. Notably, the synchronous update scheme fails to consider the variety of time scales of different processes and may cause spurious limit cycles. In order to circumvent this problem, asynchronous update schemes have been developed [23]. In deterministic asynchronous updating (DA), each network node is updated at pre-selected time intervals, corresponding to the time-scale of that particular process [24]. In the case of unknown time-scales, random order updating (ROA) or general asynchronous updating (GA) may be used. In ROA, each node is updated in each time step, but updating is carried out in a random order, whereas in GA, a randomly selected node is updated at each time step [24]. We have obtained very similar results using these two update schemes, consistent with the findings in [24], so here, we report only the results obtained by the ROA scheme. In this paper, we concentrated on GRNs of TFs, but nodes in such networks could include proteins, mRNAs and miRNAs among others.

To date a wide variety of processes in different organisms have been modelled by Boolean networks such as flower specification of *Arabidopsis thaliana* [25], [26], the cell cycle of budding yeast, fission yeast and mammals [21], [27], [28], the embryonic development of *Drosophila melanogaster* and sea urchin [29]–[31], the gene expression in the developing forebrain [32] and around the mid- and hindbrain boundary [33] during vertebrate development.

In this study, based on published connections, we have assembled a GRN of cell-fate determining TFs responsible for ventralization. Simulations of asynchronous Boolean models of the GRN gave back previously published experimental observations including cell type determination and mutation studies. Furthermore, transcriptional interactions were predicted by our simulations, one of which was validated in cell culture experiments using a promoter-reporter system.

## Materials and Methods

### Vector Construction

To construct the pGL4-Irx3 reporter, the promoter of the human Irx3 gene was amplified from genomic DNA extracted from the A431 epidermoid carcinoma cell line by PCR with primers ATACGGTACCGGTCCTCCCCAAACTTTCCC and GATTAGATCTCTCCGCGTTCGCCTATTGAT including KpnI and BglII restriction sites for directional cloning into the pGL4.12 luciferase reporter vector (Promega). Purified PCR products and a pGL4.12 vector plasmid preparation were digested with the two restriction enzymes as prescribed by the manufacturer (New England Biolabs), purified from agarose gel slices, and ligated at 4°C overnight in the presence of T4 DNA ligase (New England Biolabs). After transformation of competent Eserichia coli DH5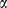 cells, transformants were grown in ampicilline containing Luria Bertani media and plasmids were purified from bacteria using the Qiagen Plasmid Mini Kit. The constructs were confirmed by bidirectional sequencing (Starseq GmbH).

To construct the pcDNA-Nkx2.2 expression vector, the cDNA of human Nkx2.2 (Source BioScience IRATp970C11116D) was amplified using PCR primers ATACAAGCTTCCACCATGTCGCTGACCAACACAAAG and GCGCCTCGAGTCACCAAGTCCACTGCTGG including HindIII and XhoI restriction sites and the Kozak sequence for optimal initiation of the translation. The PCR product was digested with the two enzymes and cloned in the eukaryotic expression vector pcDNA3 (Invitrogen) in a similar manner as described for pGL4-Irx3. All the constructs were confirmed by bidirectional sequencing (Starseq GmbH).

### Cell Culture

HEK293T human embryonic kidney cells were cultured in high-glycose DMEM (Sigma) supplemented with 10% FCS and 1% Penicillin-Streptomycin (Gibco) at 37°C in a humidified atmosphere containing 5% CO_2_. For transfections the calcium phosphate method was used as previously described [55]. Briefly, cells were seeded in pre-gelatinized 24 well plates and 24 hours after seeding, at 90% confluency, chloroquine was added to the medium. For each well of the 24-well plate, 0.83 *µg* plasmid was mixed with 6.37 *µl* 0.5 M CaCl_2_ containing solution in a total volume of 12.73 *µl*, thereafter 12.73 *µl* of HEPES buffered solution was added to the mixture of DNA and CaCl_2_, and the final solution was aerated with a pipette tip and incubated for 10 minutes before it was added to the cells dropwise. To prepare the HEPES buffered solution 28 ml 5 M NaCl solution, 11.9 g HEPES, 750 *µl* 1 M Na_2_HPO_4_ were dissolved in water to give a total volume of 500 ml and the pH was set exactly at 7.1. The plasmids used for each experimental condition (all of those repeated in quadruplicates) are indicated in table S6. The pMAX-GFP was included in varying quantities to attain a constant amount of DNA across the wells. The transfection medium containing chloroquine and calcium-phosphate precipitate was replaced by the original medium after 20 hours and the transfection efficiency was also visually controlled by observing GFP fluorescence.

### Luciferase measurement

Cells were washed in phosphate buffered saline and harvested with passive lysis buffer (Promega) for luciferase and protein measurements. Extracts were distributed into the wells of a 96 plate and Luciferase Assay Reagent II (Promega) was injected onto the samples in a Varioskan (Thermo Scientific) plate reader. The integration time was set at 1000ms for each measurement. The light emission values were normalized against the protein content of the samples measured by BCA protein assay (Thermo Scientific): 200 *µl* working reagent (50 part BCA ‘A’ +1 part BCA ‘B’) was added to 25 *µl* of each sample and after 30 minutes incubation in the dark the absorbance of the samples were measured in a Varioskan plate reader at 562 nm.

### RNA Extraction and Reverse Transcription

Total RNA from transfected cells (cells with expression of exogenous Nkx2.2: Nkx2.2_a and Nkx2.2_b) and non-transfected cells (cells which have only endogenous Nkx2.2: control_a and control_b) were isolated using the RNeasy Plus Mini kit (Qiagen, Cat. No. 74136). Following extraction, RNA Cleanup was performed with a DNase digestion step using RNeasy Mini kit (Qiagen, Cat. No. 74106) according to the manufacturers protocol. Reverse transcription was then performed by using the M-MLV reverse transcriptase (Invitrogen, Cat. No. 28025-013), with approximately 1.2 *µg* total RNA retrieved from each sample. The amplified cDNA was aliquoted and stored at −20°C.

### Real-Time PCR

The levels of Nkx2.2 gene expression were determined using relative real-time PCR quantification. Amplifications were performed with TaqMan Gene Expression Master Mix (ABI), on a Rotor-Gene Q cycler (Qiagen). Each reaction consisted of 20 ng cDNA, 1x TaqMan Gene Expression Master Mix (final concentration), 1x PrimeTime® Mini Assay probe and primer mix (IDT assay Hs.PT.49a.94096.g with 0.5 *µM* (final concentration) of each primer pair and 0.25 *µM* (final concentration) of probe). Reactions were performed in triplicate. GAPDH was used as the reference gene (IDT assay Hs.PT.49a.2918858.g). The amplification conditions comprised an initial UDG incubation at 50°C for 2 min, then denaturation at 95°C for 10 min, followed by 40 cycles of denaturation at 95°C for 15 sec, then annealing and extension at 60°C for 1 min. Sequences of primers and probes used for expression analysis are listed in Table S1. The relative expression of Nkx2.2 gene was calculated from the average Ct values of each triplicate by using the 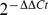 method.

### Experimental Statistics

Statistical analyses of the luciferase assay and the relative real-time PCR were performed. Differences in the levels of luciferase activity or gene expression in between samples were analysed using one-way analysis of variance (ANOVA).

### Construction of GRNs

**Figure 2:**
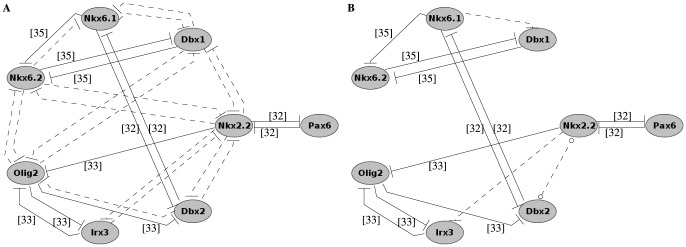
Gene regulatory networks. (A) GRN of ventralization assembled from published experimental data. TFs are denoted by ellipses. Transcriptional inhibitions between TFs and target genes are denoted by blunted arrowheads. Associations depicted by continuous lines were demonstrated experimentally; associations denoted by dotted lines were tested with Boolean simulations. (B) Minimal GRNs obtained from Boolean simulations. Inhibition represented by continuous lines were experimentally verified, additional dashed lines represent connections present in the minimal matching GRNs. The line between Nkx2.2 and Dbx2 contains a circle at both ends representing that the direction of inhibition is not defined in the minimal GRNs.

TFs downstream of Shh signalling were used as nodes of the network. Regulatory connections between them were based on published studies and possible additional links were tested using Boolean modelling (figure 2(a)).

### Conversion of a GRN to a Boolean model

The regulatory links associated with each of the GRNs shown in figure 2(a) were transformed into Boolean update rules. Only inhibitory connections were considered and hence, the general update rule for each Boolean variable 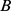 was defined as 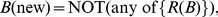
(1)where 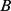 attains the values 0 or 1 and the set 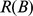 contains the Boolean values of the regulators (inhibitors) of 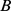. The set of inhibitors and, hence, the update rules were fixed during each Boolean simulation.

### Boolean Simulations

An asynchronous random order updating (ROA) scheme was utilized as described in [24]. Briefly, a Boolean step of ROA consists of updating all variables in a random order. All Boolean simulations were conducted from a defined initial state and at each simulation step the binary value of each variable was updated according to the corresponding Boolean logic rule. The Boolean simulations were continued until an attractor state was obtained.

### Test of alternative GRNs

For each possible GRN (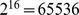 in total) shown in figure 2(a) a set of Boolean update rules were defined. For each set of update rules simulations were conducted from all possible initial states (each of the eight variables could take the value 0 or 1, so there were 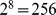 initial states). For each set of update rules a set of attractors were obtained and the fixed point attractors associated with a particular set of regulators were compared to the binary representation of the expression states of progenitor cells in the ventral neural tube (see figure 1(c)).

### Simulating knockout or overexpression

During simulations to model knockout and ectopic expression experiments, the value of the specific TF was held fixed at 0 (knockout) or 1 (ectopic expression).

## Results

### 0.1 Defining the TFs to be represented in the model

A set of TFs downstream of Shh signalling are known to promote the development of specific cell types (p3, pMN, p2, p1 and p0 neurons) in the vertebrate neural tube (see [3], [34] and references therein) as shown in figure 1(a). Combinatorial expression of eight specific TFs (namely Nkx2.2, Pax6, Olig2, Irx3, Nkx6.2, Dbx1, Nkx6.1, Dbx2) depicted in figure 1(b) determines the five distinct cell types generated in the ventral spinal cord. In our mathematical model, these TFs were chosen as variables to account for the different progenitors. Other TFs such as Nkx2.9, FoxA2 or Pax7 are also targets of Shh signalling but were not included in our model, either because of overlapping functions with other TFs or because their spatial signalling range (as defined by the location along the dorsoventral axis of the embryo) was outside of the region considered in our model.

Patterning in the neural tube is thought to be achieved via transcriptional repression [35]: Nkx2.2, Nkx6.1, Nkx6.2, Dbx1 and Dbx2, but not Pax6 or Irx3 contain a domain that has repressive effects. The inhibitory role of these TFs was demonstrated by reporter assays with full length and truncated proteins lacking the repression domain [35]. During the simulations we assumed that all factors including Pax6 and Irx3 act as transcriptional repressors, based on studies uncovering their inhibitory roles [36]–[38]. It is of interest to note that most of the TFs present in our model have been shown to act both as repressors and activators [36]–[39], their status being dependent on the presence of co-regulators [35].

In our model none of the TFs have activating inputs and hence assumed to be expressed constitutively in the absence of repressing signals. This assumption correlates well with the observation that Pax6, Irx3, Dbx1 and Dbx2 are expressed in the absence of Shh, but their expression persist even in p0 cells, when Shh is present. The other TFs in our model: Olig2, Nkx2.2, Nkx6.1 and Nkx6.2 can be activated by Shh [36]–[38], which was assumed to be present throughout the simulations. Importantly, even though Shh was not explicitely incorporated into our model, its action was represented via the assumption that the TFs activated by Shh were constitutively expressed in the absence of their inhibitors.

Interactions between the eight candidate TFs in the context of ventralization have been studied experimentally (see figure 2(a) and table S1), using a range of techniques that include analysis of protein expression patterns in either chick embryos after misexpression of TFs [37], [38] or mutant mouse embryos [38], [40].

### 0.2 Boolean network model predicts regulatory connections necessary to obtain fixed point attractors that match specific cell types in the ventral spinal cord

Developmental GRNs should be robust to perturbations [29], [41] and hence their topology is expected to define its fixed point attractors (hereafter referred to as attractors) which here correspond to experimentally observed cell phenotypes (p0, p1, p2, pMN and p3 progenitors) as shown in figure 1(c). The GRN assembled from experimentally derived regulatory connections (see the network defined by the continuous arrows in figure 2(a)) was converted into a Boolean model and asynchronous simulations were conducted (as described in Materials and Methods) to identify attractors of the system. However only cell types p0, p1 and p2 were represented and four of the obtained attractors did not correspond to any cell types (see table S2). Attractor limit cycles were not obtained in any of the simulations.

In order to obtain a GRN that can account for the different progenitor cell types that emerge during ventralization, additional regulatory connections were tested in a Boolean framework. When modelling transcriptional regulation, experimental findings (summarized in table S1) informed our modelling assumptions as follows:

- only transcriptional inhibition was considered
- transcriptional inhibition was always incorporated when there was supporting experimental evidence of regulation
- transcriptional inhibition was never incorporated when there was supporting experimental evidence of absence of regulation
- transcriptional inhibition was not incorporated if two TFs were coexpressed in at least one cell type as shown in figure 1

Using the above assumptions, the wiring diagram shown in figure 2(a), representing the interactions between the TFs, was constructed. In figure 2(a), continuous lines represent experimentally verified connections while dashed lines represent possible additional transcriptional regulations. During the simulations, the continuous lines were always present and each dashed line was either present or absent, so that in total 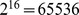 different GRNs were tested as described in Materials and Methods.

In order to obtain matching GRNs, attractors of each Boolean model were compared to the binary states that correspond to the different cell types of the ventral neural tube (see figure 1(c)). If all five cell types were represented and no additional fixed points were found, then the GRN was considered matching. The GRN containing all possible additional connections was matching, but GRNs with fewer connections were also sufficient. The two simplest GRNs, *i.e.* the GRNs containing the smallest number of regulatory connections are depicted in figure 2(b). The minimal number of additional connections compared to default was three: in all matching GRNs Nkx6.1 was predicted to inhibit Dbx1 and Nkx2.2 was predicted to inhibit Irx3; additionally, in the two simplest matching GRNs, either Nkx2.2 inhibited Dbx2 or Dbx2 inhibited Nkx2.2.

**Figure 3:**
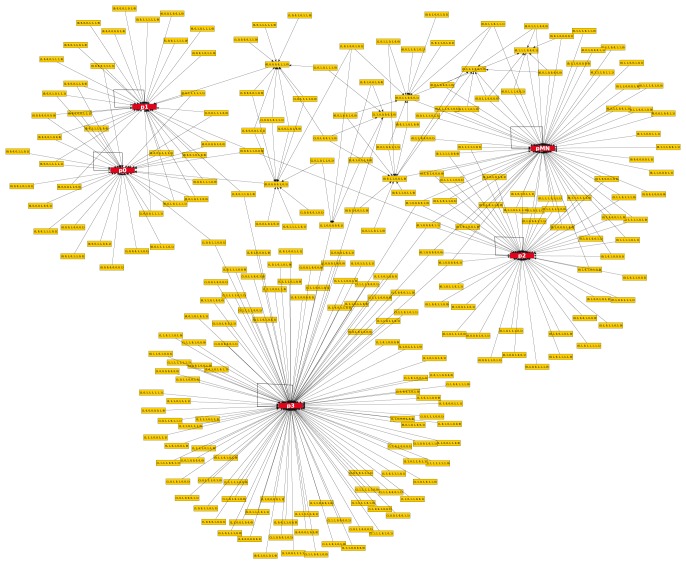
Boolean simulation results of the minimal GRN where Nkx2.2 inhibits Dbx2. In contrast to figure 2, the nodes in this figure represent the TF expression states as a vector of zeros and ones in the order (Nkx2.2, Nkx6.1, Olig2, Pax6, Irx3, Dbx2, Nkx6.2, Dbx1). Red nodes stand for attractor states and are denoted by the name of the corresponding neural progenitor (p0, p1, p2, pMN and p3 cells), other nodes are labelled by the expression level of each transcription factor. As a result of the asynchronous update in the Boolean model, one state can have more than one successor states, but the figure only shows the most probable update route(s) for each node.

Next, we studied the dynamics of the simplest matching GRNs. Figure 3 shows the results obtained with ROA updating (see Materials and Methods) and using the Boolean logic rules corresponding to the GRN depicted in figure 2(b) when Nkx2.2 inhibits Dbx2. Unlike figure 2, where nodes represent TFs, the nodes in figure 3 represent the joint expression states of TFs, *i.e.* the binary value of each TF in the order (Nkx2.2, Nkx6.1, Olig2, Pax6, Irx3, Dbx2, Nkx6.2, Dbx1). Red nodes indicate attractor states and are denoted by the name of the corresponding neural progenitor (p0, p1, p2, pMN and p3 cells), other nodes are labelled by the expression level of each TF. Because of the asynchronous updating, one state can have more than one successor states, but attractors of the system persist. In figure 3, edges starting from a node represent the most likely update route: in the case of two or more arrows starting from the same node, more than one successor states were equally possible.

The full transition tables for both of the simplest Boolean logic rules are shown in tables S3 and S4. In the case of the simplest logic rule, when Nkx2.2 inhibits Dbx2, transient oscillations never occur and starting from any of the initial states, an attractor state may be reached in a single Boolean step. When Dbx2 inhibits Nkx2.2, transient oscillations may occur and starting from any of the initial states, the minimum number of Boolean steps to reach a steady state is one or two.

For each attractor the possible basin of attraction [42] is defined as the set of input nodes from which at least one possible path leads into the attractor state. The percentage of the basin of attraction was calculated for the nodes representing p0, p1, p2, pMN and p3 cells as a percentage of the initial nodes that can attain the attractor. The weighted percentage of the basin of attraction was also calculated: the contribution of each initial state was divided by the number of attractors it could attain. In the simplest GRN when Nkx2.2 inhibits Dbx2, the basin of attraction (see table S5) is largest for the most ventral p3 state and gradually decreases towards attractors corresponding to more dorsal cell phenotypes, whereas when Dbx2 inhibits Nkx2.2 the basin of attraction is largest for the pMN and only the second largest for p3. This result suggests, that the p3 or pMN phenotype is the most robust and the p0 and p1 are the least robust cell types.

The regulatory connections hypothesized by the model were examined further. Each of the inhibition Nkx2.2
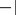
Dbx2 and Dbx2
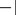
Nkx2.2 was only present in one of the simplest GRNs found by Boolean modelling (figure 2(b)) and as such their likelihood of being a genuine link is smaller than the others. The inhibitions Nkx6.1
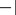
Dbx1 and Nkx2.2
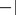
Irx3 both represent regulatory connections between TFs expressed in distant progenitor domains and, to the best of our knowledge neither link has been validated experimentally yet. Even so, some experimental evidence is consistent with the predicted Nkx6.1
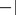
Dbx1 link: the findings that Dbx1 is repressed by Nkx6.2 [40] and that Nkx6.1 and Nkx6.2 show overlapping functions [40] can be taken together to indicate a possible Nkx6.1
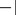
Dbx1 inhibition. We note that this regulatory link was hypothesized by Vallstedt et al. (see figure 8(b) in [40]). To the best of our knowledge not even such an indirect evidence for Nkx2.2
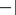
Irx3 yet exists and so in section 0.4 we focused our attention to verify the hypothesis that Nkx2.2 inhibits Irx3.

### 0.3 The Boolean model reproduces experimental results involving over- and underexpression of specific genes

Although the dynamic development of different cell types as a function of Shh signalling could not be studied by our model, it was possible to predict the response to changes in TF expressions. In order to check the validity of our model predictions, under- and over-expression studies were replicated *in silico*. In the simulations the binary representation of the different neuronal cell phenotypes (see figure 1 (c)) determined the initial values of each TF and the variables were updated at each time step according to the Boolean rules corresponding to the simplest GRNs shown in figure 2(b) (both rules yielded the same results). Under-(over-)expression was simulated by holding the value of the relevant variable fixed at zero (one) and the simulation was continued until an attractor was reached.

We considered experiments in which motoneuron (MN) progenitors were converted from or to different cell types. We chose this cell type for further study because of its outstanding clinical significance as the progenitor which gives rise to MNs that directly innervate muscles. The *in silico* simulation results are summarized in table 1 and discussed below. The first columns in table 1 show the simulation details: initial state, the list of variables kept fixed at 0 or 1 and the attractor obtained. As a result of the asynchronous updating, more than one attractor is possible. The last two columns show the reference paper and compare the simulation results with the published experimental results.

**Table 1:** Boolean simulation results conducted with the given initial state and values kept fixed at 0 (knockout genes) or at 1 (overexpressed genes) to reproduce experimental results.

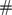	initial state	knockout	overexpression	attractor	experiment	reproduced?
1	p0, p1, p2	Irx3	Nkx6.1	pMN	[37] fig. 5 (a)	yes
2	p0, p1	-	Nkx6.1	p2	[37] fig. 5 (b)	yes
3	pMN	-	Irx3	p2	[37] fig. 6	yes
4	pMN	-	Nkx2.2	p3	[37] fig. 7	yes
5	pMN	Nkx6.1	-	(0, 0, 1, 1, 0, 0, 1, 0)	[43] figs. 2, 4	yes
				or (0, 0, 1, 1, 0, 0, 0, 1)		
6	p2	Nkx6.1	-	p1 or p0	[43] fig. 4	partly
7	pMN	Nkx6.1, Nkx6.2	-	(0, 0, 1, 1, 0, 0, 0, 1)	[40] fig. 6	partly
8	p2	Nkx6.1, Nkx6.2	-	p0	[40] fig. 6	yes
9	p0, p1, p2	-	Olig2	pMN	[38] fig. 4	yes

Briscoe et al. studied the results of misexpression of Nkx6.1 [37] in cells dorsal to the MN domain. MN subtype determinant markers were detected when Nkx6.1 was misexpressed in the absence of high-level Irx3 expression (in cells where expression of Nkx6.1 occurred earlier than Irx3), whereas V2 neuron markers were detected when co-expression of Nkx6.1 and Irx3 occurred. They also studied Irx3 and Nkx2.2 misexpression in MN progenitors [37]. In Irx3 expressing cells MN markers were absent and V2 neuronal markers were detected more ventral to normal positions. Similarly, MN markers were repressed in Nkx2.2 expressing cells and V3 neuronal markers appeared.

*In silico* Boolean simulations could reproduce the effect of Nkx6.1 overexpression (simulation 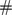
1 in table 3) when Irx3 was absent (Irx3 = 0 throughout the simulation). Conversely, cells were predicted to attain the p2 and not the pMN state, when Irx3 expression levels were allowed to change (simulation 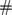
2 in table 3). Although this result did not explain how Irx3 is inhibited during the initial phase of progenitor specification, it showed that in order to obtain pMN cells, the final progenitor cell phenotype had to be attained before Irx3 expression started, as otherwise cells attained the p2 neuronal phenotype. This result is consistent with the experimental observation reported in [37]. Simulations yielded p2 and p3 cell types when Irx3 and Nkx2.2 were fixed respectively at 1 (see simulations 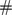
3, 4) in agreement with the experimental results [37].

When Sander et al. studied expressions of neural progenitor markers in Nkx6.1 mutant mouse embryos, they found that in these mice p2 progenitor cells switched fate to p1 cells [43] and MN cell development was also disrupted [43]. The Boolean model could partially reproduce these findings: when we fixed Nkx6.1 = 0, pMN cells turned into an unidentified cell type and p2 cells into p1 or p0 (simulations 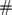
5, 6).

In the case of Nkx6.1, Nkx6.2 double mutants, ectopic expression of p0 marker was found in p2 and pMN domains, suggesting that some of the cells that would have originally developed to p2 or pMN, turned into p0 [40]. In Boolean simulations, with Nkx6.1 = Nkx6.2 = 0, pMN cells turned into an unidentified cell type and p2 cells into p1 (simulation 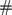
7, 8), so that once again the experimental findings were partly reproduced.

Novitch et al. studied the effect of misexpressed Olig2 in the spinal cord and found MN markers in cells that expressed Olig2 [38]. This experiment was simulated by keeping the value of Olig2 fixed at 1 in p2, p1 and p0 cells. In all cases, the obtained attractor corresponded to the pMN cell type as summarized in simulation 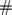
9.

### 0.4 Nkx2.2 represses the transcriptional activity of the Irx3 promoter in a cellular context

Boolean modelling of the GRN of ventralization predicts that an inhibitory link between Nkx2.2 and Irx3 is necessary to attain all attractors associated with the different neural progenitor cell phenotypes. To validate the predicted Nkx2.2
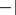
Irx3 link experimentally, we have constructed a reporter plasmid (hereafter referred to as pGL4-Irx3) based on the pGL4.12 reporter vector (Promega) containing an improved luciferase reporter gene. The promoter of the Irx3 gene (chr16:54321583-54323661) was amplified by PCR from genomic DNA extracted from the A431 [44] human epidermic carcinoma cell line and inserted upstream of the cDNA encoding luciferase. To express Nkx2.2, its full length cDNA was ordered from SourceBioscience (IRATp970C11116D), the appropriate restriction sites were added by PCR amplification and it was cloned it into the eukaryotic expression vector pcDNA3 (Invitrogen).

**Figure 4:**
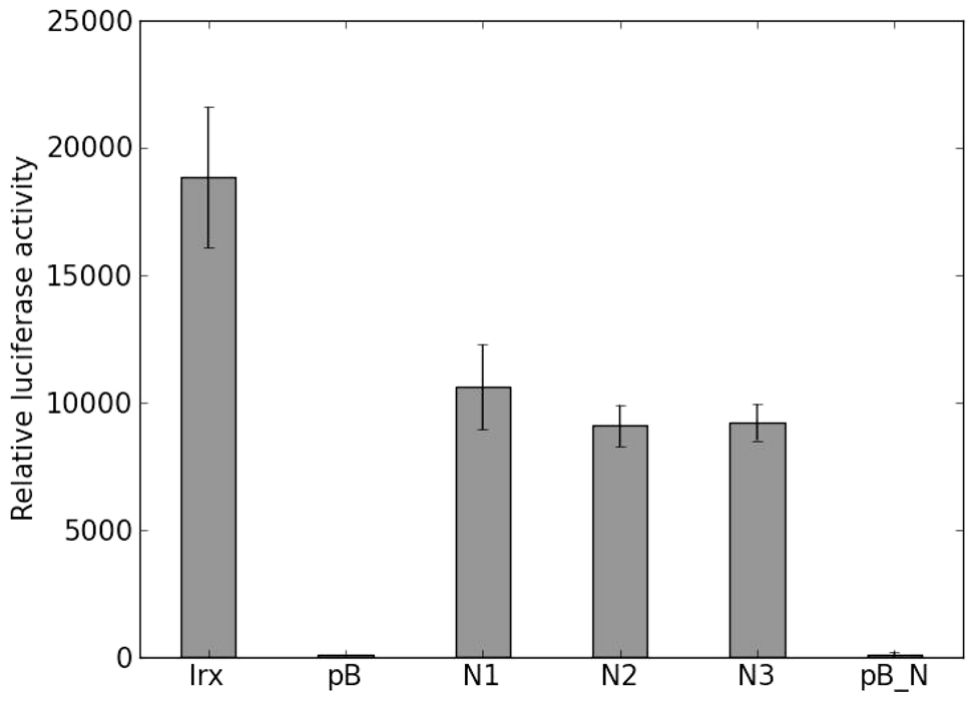
Relative luciferase activity. The light emission of luciferase was measured after addition of Luciferase Assay Reagent II, normalized against the protein content in samples transfected by pGL4-Irx3 (Irx), promoterless pGL4.12 (pB), pGL4-Irx3 plus increasing molar ratio of Nkx2.2 (N1, N2, N3) and promoterless pGL4.12 plus Nkx2.2. In the promoterless reporter vectors there is virtually no activity. The luciferase activity in samples N1, N2, N3 significantly differ from that of sample Irx3 (significance level 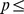
0.05). The reduction of the luciferase activity shows that Nkx2.2 negatively regulates Irx3 promoter activity, however no dose-dependent inhibition was observed.

**Figure 5:**
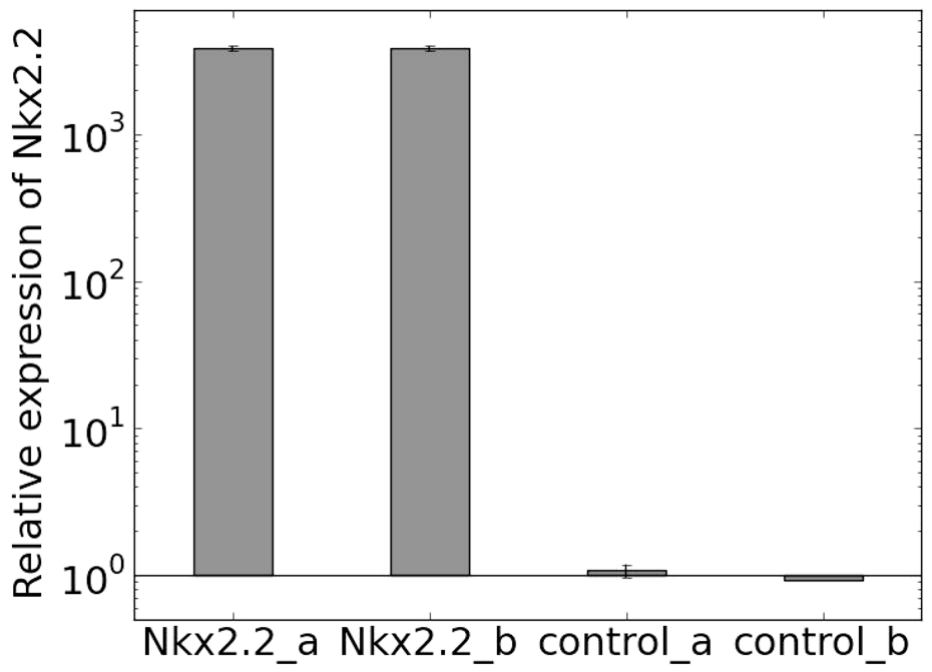
Relative expression of Nkx2.2 in the transfected cells (cells with exogenous expression of Nkx2.2: Nkx2.2_a and Nkx2.2_b) and non-transfected cells (cells which have only endogenous expression of Nkx2.2: control_a and control_b). Relative expression was calculated using the 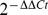 method. Ct values were normalized to the reference gene GAPDH and the average expression level of the non-transfected cells (control_a, control_b) were normalized to 1. Expression level of Nkx2.2 in transfected cells significantly differ from expression level of Nkx2.2 in non-transfected cells (significance level 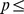
0.05).

Calcium-phosphate mediated transient co-transfection experiments were performed in HEK293T [45] cells. The pGL4-Irx3 construct or a promoterless pGL4.12 was introduced into the cells with or without different amounts of the pcDNA3-Nkx2.2 plasmid (see figure 4(a)). Green fluorescent protein (GFP) expressing control plasmid, that does not bind to or regulate the expression of Irx3, was spiked onto every condition to verify the transfection efficiency (see a representative photo of transfected cells in figure S1) while the total amount of DNA and the amount of pGL4-Irx3 or promoterless pGL4.12 reporter plasmid were kept constant. Additionally, qPCR was used to confirm that the transfected cells expressed exogenous Nkx2.2 (see figure 5). The amount of cotransfected plasmids in each condition for qPCR and luciferase measurements are listed in table S6.

We found that the transcriptional activity of the Irx3 promoter was significantly reduced in the presence of Nkx2.2 (figure 4). This observation provides compelling evidence that Nkx2.2 can regulate the Irx3 promoter in a cellular context. Of note, TLE-family proteins can act as co-repressors for Nkx2.2 [35], and TLE-like activity has already been reported in HEK293T cells [46]. Therefore the finding that Nkx2.2 acted in these experiments as a repressor and not as an activator was in line with our expectations. Importantly, we could not observe any effect of Nkx2.2 on the promoterless pGL4.12 vector that itself had a very low level of activity (figure 4), presumably due to the removal of cryptic TF binding sites from its backbone by the manufacturer. The lack of Nkx2.2 effect on the empty pGL4.12 further corroborates that the transcriptional change we have measured is attributable to an effect on the Irx3 promoter sequence and not on any other element in the pGL4-Irx3 plasmid.

Of note, the degree of inhibition induced by different amounts of Nkx2.2 did not differ significantly (see figure 4). The absence of a graded response suggests that the maximal effect was shown, since higher Nkx2.2 concentrations did not induce increased inhibition, indicating that in this concentration range of Nkx2.2 the system may be accurately modelled using a Boolean approach in which Irx3 expression levels are either on or off.

## Discussion

We have presented a Boolean model of the GRN of ventralization that could attain five different steady states corresponding to five different neuronal progenitor cells types in the ventral spinal cord, more than any of the previous models [13], [15], [17], [18]. The dynamic evolution of three different cell types (p2, pMN, p3) was captured by the GRN model first presented in [17] and later further analysed in [18] (see figure 4 in [17]), with model variables Gli, Pax6, Olig2 and Nkx2.2. In that model, the level of Gli serves as an input and captures the effect of Shh signalling while the levels of other TFs define the cell type. In our GRN (figure 2(b)) the subnetwork formed by Pax6, Olig2 and Nkx2.2 differs from that in [17], [18], because the repressive connections Olig2
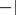
Nkx2.2 and Olig2
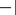
Pax6 are absent.

We did not consider Nkx2.2 inhibition by Olig2, because Novitch et al. have shown experimentally by misexpressing Olig2, that it does not regulate Nkx2.2 [38]. Note however, that Balaskas et al. found the opposite, *i.e.* that overexpression of Olig2 inhibited Nkx2.2 and in Olig2^−/−^ chick embryos a low level of dorsal expression of Nkx2.2 was observed [17]. These experimental results contradict each other, and our model predictions do not favour one or the other either: all of the neuronal progenitor cell types considered could be attained with or without the Olig2
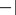
Nkx2.2 link.

In our model, Pax6 inhibition by Olig2 was also ignored because both TFs are expressed in pMN cells. This regulatory link could be included in the model *i.e.* by assuming three expression states of Pax6 (0, 1, 2), where inhibition of Olig2 would result in decreased but not zero expression of Pax6. In fact, experimental results suggest ternary Pax6 expression in the ventral neural tube in contrast to the binary regulation of Nkx2.2 (see figure 2 in [36]). Graded expression of Pax6 would only make a difference in the simulation results if Pax6 regulated more than one targets at different expression levels. In order to assess whether including the Olig2
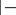
Pax6 link would predict different regulatory links compared to those shown in figure 2(b), we have repeated the simulations described in section 0.2. During the simulations, continuous lines shown in figure 2(a) and the Olig2
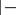
Pax6 link were always present and each dashed line was either present or absent. Attractors in each simulation were compared to the binary representation of the cell types supplemented with the ternary Pax6 levels (see figure S2). However, the necessary additional links were found to be the same, indicating that even if transcriptional connections may be missing from our GRNs, the Boolean simulations still allow to predict indispensable inhibitory connections.

Opposed to the conventional models of morphogen patterning where TFs are thought to be regulated at different morphogen levels, in the case of Shh signalling Cohen et al. describe another possible scenario, where gene expression is determined by the combined effect of Gli proteins and a cross-repressive GRN [9]. In order to describe the effects of the morphogen signalling, both building blocks (*i*.e. the dynamics of Gli proteins as a function of Shh and the topology of the GRN) needs to be characterized. Lai et al. have presented a model of the Shh signalling network, which predicts the temporal response of Gli proteins as a function of Shh. In the current paper, Boolean modelling was applied to predict regulatory links between TFs based on the attractor states of the model, and hence provide a circuitry for the GRN of ventralization. Combining these models would potentially provide a temporal response of the TFs in the GRN as a function of Shh signalling, and could describe the dynamic emergence of different neuronal progenitor phenotypes. In this paper we used a Boolean strategy to predict regulatory links between TFs based on the attractor states of the model. For a given regulatory logic, ODE modelling could then be employed to further characterise the GRN such as shown in [17], [18].

The minimal GRNs obtained with our Boolean simulations have suggested that a regulatory connection exists between Nkx2.2 and Irx3, a prediction that has been tested experimentally. Nkx2.2 can exhibit transcriptional activation or repression based on the presence of coregulators [35], [39]. We have found that Nkx2.2 decreased Irx3 promoter activity in line with the previous observation that TLE like corepressor activity has been reported in HEK293T cells [46]. However, clarifying which one of TLE proteins or what combination of them is responsible for the repressive nature of Nkx2.2 in HEK293T cells would necessitate further studies.

While to the best of our knowledge, a direct connection between Nkx2.2 and Irx3 has not previously been mentioned in the literature, the existence of such a connection can be inferred from some published results. For example, images of the developing spinal cord of Olig2 knock-out mice reveal a sharp boundary between the Irx3 and Nkx2.2 expressing cells with virtually no cells expressing both factors [47], [48]. It can be hypothesized that, under physiological conditions, the inhibition of Irx3 by Nkx2.2 has a functional role in the genetic regulatory network, because at least some p3 neurons first express the motoneuron marker Olig2, and Nkx2.2 accumulates after Olig2 has been downregulated [4]. Nkx2.2 activity in these cells may help to maintain the Irx3 gene in a repressed state.

The connection between Nkx2.2 and Irx3 could be of physiological significance in tissues other than the central nervous system. Interestingly, in pancreatic development, the absence of Nkx2.2 produces a phenotype in mice [39] that is similar to that seen in zebrafish that lack Irx3 function [49]: in both cases the insulin producing *β*-cells are absent, and the number of glucagon-producing *α*-cells is markedly reduced. These observations are consistent with a direct interaction between Nkx2.2 and Irx3 only if Nkx2.2 activates Irx3. However, at least in *α*-cells and in uncommitted ghrelin producing cells, such relationship is not excluded in the light of the observation that Nkx2.2 can activate transcription [39]. Evenso, the absence of *β*-cells requires a more complex interpretation since Nkx2.2 can play an inhibitory role in *β*-cells through its recruitment of TLE-3 (Grg-3) to its Tinman-domain and such inhibition is crucial for establishing the identity of *β*-cells [50]. Of note, microarray experiments have shown that, Irx3 expression is downregulated in the pancreas of Nkx2.2-null mice at embryonic day 12.5 [51].

The ability of Nkx2.2 to regulate Irx3 can also be important in forebrain development. In the diencephalon, sonic hedgehog signalling escalates dorsally along two thin lines called zona limitans intrathalamica (ZLI). Nkx2.2 is induced both rostrally and ventrally from the dorsoventrally stretched Shh signalling centre of the ZLI, so that the Nkx2.2 positive regions from both sides of the embryo touch at the dorsal pole to form a closed, ring-shaped area that surrounds the diencephalon [52]. Pax6 is expressed rostrally while Irx3 appears exclusively caudally from this area. Further, forced expression of Irx3 in cells anterior to the ZLI results in the up-regulation of genes that otherwise define posterior fate [53]. Therefore we hypothesise that Nkx2.2 expression along the ZLI contributes to the creation of a sharp boundary of Irx3 expression via its repressor effect on Irx3 as described in this study. Although Irx3 is considered to be a prepattern gene in this brain area and its global landscape of expression is not changed when Shh signalling is inhibited in ZLI, some Irx3 positive cells can be observed anterior to the boundary when Shh signal transduction is blocked [54].

## Conclusions

In this study we have employed a Boolean strategy to map the transcriptional connections that regulate ventralization. The minimal GRN resulting from this approach accounted for five different neural progenitor cell types and it could reproduce effects of perturbations in expression levels of certain TFs observed experimentally. The power of this approach was demonstrated when one of the predicted regulatory connections was verified experimentally using a promoter reporter assay. Our finding that Nkx2.2 transcriptionally inhibits Irx3 could be important in a wider context playing a role in the development of the pancreas and forebrain. This illustrates how the application of mathematical models developed to describe a specific system can yield useful insight and generate new testable predictions in other areas as well.

## Supporting Information

Figure S1 **Transcription efficiency in HEK293T by fluorescent imaging of GFP-expression.** There was no fluorescent signal in the negative control condition. Brightfield image (a) and fluorescent image (b) of the same visual field at 200x magnification of a representative sample.
(PDF)

Figure S2
**Gene regulatory networks.** Ternary representation of Pax6 and binary representation of the other TFs in each progenitor cell type in the order (Nkx2.2, Nkx6.1, Olig2, Pax6, Irx3, Dbx2, Nkx6.2, Dbx1) (a). The default GRN shown in figure 2(a) supplemented with an Olig2
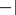
Pax6 link (b) and the minimal GRN obtained from it (c). The dashed-dotted link represents that Olig2 inhibition only reduces Pax6 expression state to 1, but not to 0. Contrary to this, Nkx2.2 completely inhibits Pax6 expression. Dashed lines represent connections experimentally not verified but present in the minimal matching GRN. The line between Nkx2.2 and Dbx2 contains a circle at both ends representing that the direction of inhibition is not defined (cf. figure 2).(PDF)

Table S1 **Inhibitory links between transcription factors (TFs) based on experimental studies.** References correspond to confirmed connections or the absence of them. We assumed the absence of transcriptional inhibition if two TFs were coexpressed in at least one neural progenitor cell type: this information is also depicted.
(PDF)

Table S2 **Boolean attractor states.** Binary representation of the attractors states of the Boolean model of the GRN assembled from literature results (see figure 2(a)) in the order (Nkx2.2, Nkx6.1, Olig2, Pax6, Irx3, Dbx2, Nkx6.2, Dbx1) and the corresponding neural progenitor cell type if it exists (cf. figure 1). The attractor states do not account for pMN and p3 cell types and some of the attractors do not correspond to existing cell types.
(PDF)

Table S3 **Transition probability between TF expression states when Nkx2.2 inhibits Dbx2.** For each possible input node (row names) displays the probability of a transition into an output node (column names) as a result of a Boolean step. In the simulations, the Boolean update rule corresponding to the GRN depicted in figure 2(b) when Nkx2.2 inhibits Dbx2 was used.
(XLSX)

Table S4 **Transition probability between TF expression states when Dbx2 inhibits Nkx2.2.** For each possible input node (row names) displays the probability of a transition into an output node (column names) as a result of a Boolean step. In the simulations, the Boolean update rule corresponding to the GRN depicted in figure 2(b) when Dbx2 inhibits Nkx2.2 was used.
(XLSX)

Table S5 **Normal and weighted percentage of the possible basin of attraction for each attractor state of the Boolean model of the minimal GRN shown in** **figure 2(b)** **when Nkx2.2 inhibits Dbx2 (a) and when Dbx2 inhibits Nkx2.2 (b).** The percentage of the basin of attraction was calculated as the number of initial states that can attain the attractor divided by the total number of initial states. As a result of the asynchronous update rules, one initial state could attain more than one attractors. In order to calculate the weighted percentage of the basin of attraction, the contribution of each initial state was divided by the number of attractors it could attain.
(PDF)

Table S6 **DNA content in CaCl_2_ transfected cells for luciferase (A) and qPCR (B) measurements.** HEK293T cells were transfected with different amounts of plasmids to conduct qPCR and luciferase measurements (see results in figures 4 and 5). The indicated molar ratio of each plasmid was added to the transfection medium for each condition.
(PDF)
