# Supplementary material for: Boolean Modelling Reveals New Regulatory Connections between Transcription Factors Orchestrating the Development of the Ventral Spinal Cord
Source: PLoS One. 2014 Nov 14;9(11):e111430. doi: 10.1371/journal.pone.0111430 (PMC4232242; doi:10.1371/journal.pone.0111430)
Supplement: Figure S1 — Transcription efficiency in HEK293T by fluorescent imaging of GFP-expression. There was no fluorescent signal in the negative control condition. Brightfield image (a) and fluorescent image (b) of the same visual field at 200x magnification of a representative sample. (PDF) [file pone.0111430.s001.pdf]

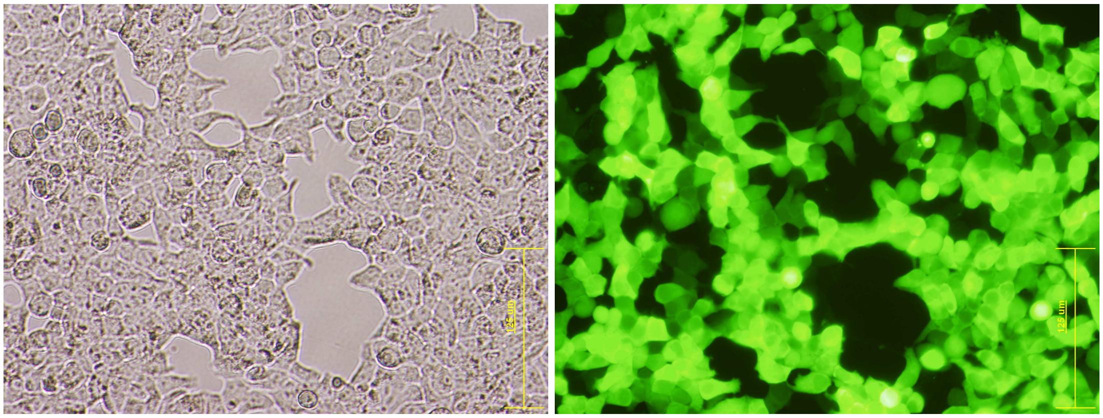

Figure S-1 Transcription efficiency in HEK293T by fluorescent imaging of GFP-expression. There was no fluorescent signal in the negative control condition. Brightfield image (a) and fluorescent image (b) of the same visual field at 200x magnification of a representative sample.
