# Supplementary material for: Boolean Modelling Reveals New Regulatory Connections between Transcription Factors Orchestrating the Development of the Ventral Spinal Cord
Source: PLoS One. 2014 Nov 14;9(11):e111430. doi: 10.1371/journal.pone.0111430 (PMC4232242; doi:10.1371/journal.pone.0111430)
Supplement: Figure S2 — Gene regulatory networks. Ternary representation of Pax6 and binary representation of the other TFs in each progenitor cell type in the order (Nkx2.2, Nkx6.1, Olig2, Pax6, Irx3, Dbx2, Nkx6.2, Dbx1) (a). The default GRN shown in figure 2(a) supplemented with an Olig2Pax6 link (b) and the minimal GRN obtained from it (c). The dashed-dotted link represents that Olig2 inhibition only reduces Pax6 expression state to 1, but not to 0. Contrary to this, Nkx2.2 completely inhibits Pax6 expression. Dashed lines represent connections experimentally not verified but present in the minimal matching GRN. The line between Nkx2.2 and Dbx2 contains a circle at both ends representing that the direction of inhibition is not defined (cf. figure 2). (PDF) [file pone.0111430.s002.pdf]

cell type	binary representation (Nkx2.2, Nkx6.1, Olig2, Pax6, Irx3, Dbx2, Nkx6.2, Dbx1)
P0	(0, 0, 0, 2, 1, 1, 0, 1)
P1	(0, 0, 0, 2, 1, 1, 1, 0)
P2	(0, 1, 0, 2, 1, 0, 0, 0)
PMN	(0, 1, 1, 1, 0, 0, 0, 0)
P3	(1, 1, 0, 0, 0, 0, 0, 0)

(a)

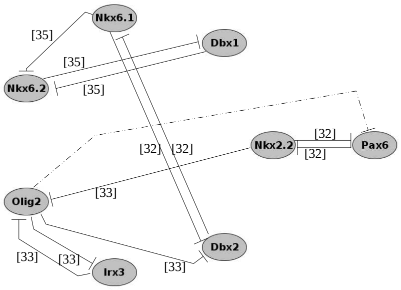

(b)

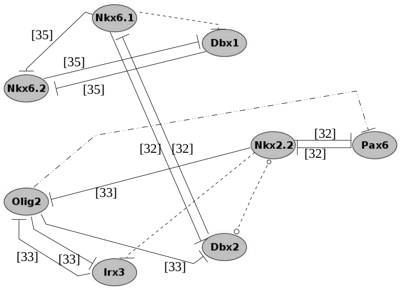

(c)

Figure S-1 Gene regulatory networks. Ternary representation of Pax6 and binary representation of the other TFs in each progenitor cell type in the order (Nkx2.2, Nkx6.1, Olig2, Pax6, Irx3, Dbx2, Nkx6.2, Dbx1) (a). The default GRN shown in figure 2(a) supplemented with an Olig2$-|$Pax6 link (b) and the minimal GRN obtained from it (c). The dashed-dotted link represents that Olig2 inhibition only reduces Pax6 expression state to 1, but not to 0. Contrary to this, Nkx2.2 completely inhibits Pax6 expression. Dashed lines represent connections experimentally not verified but present in the minimal matching GRN. The line between Nkx2.2 and Dbx2 contains a circle at both ends representing that the direction of inhibition is not defined (cf. figure 2).
