# Supplementary material for: Boolean Modelling Reveals New Regulatory Connections between Transcription Factors Orchestrating the Development of the Ventral Spinal Cord
Source: PLoS One. 2014 Nov 14;9(11):e111430. doi: 10.1371/journal.pone.0111430 (PMC4232242; doi:10.1371/journal.pone.0111430)
Supplement: Table S1 — Inhibitory links between transcription factors (TFs) based on experimental studies. References correspond to confirmed connections or the absence of them. We assumed the absence of transcriptional inhibition if two TFs were coexpressed in at least one neural progenitor cell type: this information is also depicted. (PDF) [file pone.0111430.s003.pdf]

target	regulator	inhibition	experimental evidence	target	regulator	inhibition	experimental evidence
Nkx2.2	Nkx6.1	NO	coexpressed	Irx3	Nkx2.2	?	
	Olig2	NO	Novitch et al. [1]		Nkx6.1	NO	coexpressed
	Pax6	YES	Briscoe et al. [2]		Olig2	YES	Novitch et al. [1]
	Irx3	?			Pax6	NO	coexpressed
	Dbx2	?			Dbx2	NO	coexpressed
	Nkx6.2	?			Nkx6.2	NO	coexpressed
	Dbx1	?			Dbx1	NO	coexpressed
Nkx6.1	Nkx2.2	NO	Briscoe et al. [2]	Dbx2	Nkx2.2	?	
	Olig2	NO	Novitch et al. [1]		Nkx6.1	YES	Briscoe et al. [2]
	Pax6	NO	coexpressed		Olig2	YES	Novitch et al. [1]
	Irx3	NO	coexpressed		Pax6	NO	Briscoe et al. [2]
	Dbx2	YES	Briscoe et al. [2]		Irx3	NO	coexpressed
	Nkx6.2	?			Nkx6.2	NO	coexpressed
	Dbx1	?			Dbx1	NO	coexpressed
Olig2	Nkx2.2	YES	Novitch et al. [1]	Nkx6.2	Nkx2.2	?	
	Nkx6.1	NO	Novitch et al. [1]		Nkx6.1	YES	Vallstedt et al. [3]
	Pax6	NO	coexpressed		Olig2	?	
	Irx3	YES	Novitch et al. [1]		Pax6	NO	coexpressed
	Dbx2	?			Irx3	NO	coexpressed
	Nkx6.2	?			Dbx2	NO	coexpressed
	Dbx1	?			Dbx1	YES	Vallstedt et al. [3]
Pax6	Nkx2.2	YES	Briscoe et al. [2]	Dbx1	Nkx2.2	?	
	Nkx6.1	NO	coexpressed		Nkx6.1	?	
	Olig2	NO	coexpressed		Olig2	?	
	Irx3	NO	coexpressed		Pax6	NO	coexpressed
	Dbx2	NO	coexpressed		Irx3	NO	coexpressed
	Nkx6.2	NO	coexpressed		Dbx2	NO	coexpressed
	Dbx1	NO	coexpressed		Nkx6.2	YES	Vallstedt et al. [3]

Table S-1 Inhibitory links between transcription factors (TFs) based on experimental studies. References correspond to confirmed connections or the absence of them. We assumed the absence of transcriptional inhibition if two TFs were coexpressed in at least one neural progenitor cell type: this information is also depicted.

References

[1] Novitch B, Chen A, Jessell T (2001) Coordinate regulation of motor neuron subtype identity and pan-neuronal properties by the bHLH repressor Olig2. Neuron 31: 773–789.

[2] Briscoe J, Pierani A, Jessell T, Ericson J (2000) A homeodomain protein code specifies progenitor cell identity and neuronal fate in the ventral neural tube. Cell 101: 435–445.

[3] Vallstedt A, Muhr J, Pattyn A, Pierani A, Mendelsohn M, et al. (2001) Different levels of repressor activity assign redundant and specific roles to Nkx6 genes in motor neuron and interneuron specification. Neuron 31: 743–755.
