# Supplementary material for: Boolean Modelling Reveals New Regulatory Connections between Transcription Factors Orchestrating the Development of the Ventral Spinal Cord
Source: PLoS One. 2014 Nov 14;9(11):e111430. doi: 10.1371/journal.pone.0111430 (PMC4232242; doi:10.1371/journal.pone.0111430)
Supplement: Table S2 — Boolean attractor states. Binary representation of the attractors states of the Boolean model of the GRN assembled from literature results (see figure 2(a)) in the order (Nkx2.2, Nkx6.1, Olig2, Pax6, Irx3, Dbx2, Nkx6.2, Dbx1) and the corresponding neural progenitor cell type if it exists (cf. figure 1). The attractor states do not account for pMN and p3 cell types and some of the attractors do not correspond to existing cell types. (PDF) [file pone.0111430.s004.pdf]

Attractor state	cell type
(0, 0, 0, 1, 1, 1, 1, 0)	p1
(1, 0, 0, 0, 1, 1, 0, 1)	-
(1, 0, 0, 0, 1, 1, 1, 0)	-
(0, 1, 0, 1, 1, 0, 0, 1)	p2
(0, 1, 1, 1, 0, 0, 0, 1)	-
(0, 0, 0, 1, 1, 1, 0, 1)	p0
(1, 1, 0, 0, 1, 0, 0, 1)	-

Table S-1 Boolean attractor states. Binary representation of the attractors states of the Boolean model of the GRN assembled from literature results (see figure 2(a)) in the order (Nkx2.2, Nkx6.1, Olig2, Pax6, Irx3, Dbx2, Nkx6.2, Dbx1) and the corresponding neural progenitor cell type if it exists (cf. figure 1). The attractor states do not account for pMN and p3 cell types and some of the attractors do not correspond to existing cell types.
