# Supplementary material for: Boolean Modelling Reveals New Regulatory Connections between Transcription Factors Orchestrating the Development of the Ventral Spinal Cord
Source: PLoS One. 2014 Nov 14;9(11):e111430. doi: 10.1371/journal.pone.0111430 (PMC4232242; doi:10.1371/journal.pone.0111430)
Supplement: Table S5 — Normal and weighted percentage of the possible basin of attraction for each attractor state of the Boolean model of the minimal GRN shown in figure 2(b) when Nkx2.2 inhibits Dbx2 (a) and when Dbx2 inhibits Nkx2.2 (b). The percentage of the basin of attraction was calculated as the number of initial states that can attain the attractor divided by the total number of initial states. As a result of the asynchronous update rules, one initial state could attain more than one attractors. In order to calculate the weighted percentage of the basin of attraction, the contribution of each initial state was divided by the number of attractors it could attain. (PDF) [file pone.0111430.s007.pdf]

Attractor	Basin of attraction	
	percentage	weighted %
p0	0.39	0.10
p1	0.39	0.10
p2	0.56	0.17
pMN	0.63	0.23
p3	0.75	0.40

(a)

Attractor	Basin of attraction	
	percentage	weighted %
p0	0.39	0.10
p1	0.39	0.10
p2	0.56	0.17
pMN	0.63	0.23
p3	0.75	0.40

(b)

Table S-1 Normal and weighted percentage of the possible basin of attraction for each attractor state of the Boolean model of the minimal GRN shown in figure 2(b) when Nkx2.2 inhibits Dbx2 (a) and when Dbx2 inhibits Nkx2.2 (b). The percentage of the basin of attraction was calculated as the number of initial states that can attain the attractor divided by the total number of initial states. As a result of the asynchronous update rules, one initial state could attain more than one attractors. In order to calculate the weighted percentage of the basin of attraction, the contribution of each initial state was divided by the number of attractors it could attain.
