# Supplementary material for: Boolean Modelling Reveals New Regulatory Connections between Transcription Factors Orchestrating the Development of the Ventral Spinal Cord
Source: PLoS One. 2014 Nov 14;9(11):e111430. doi: 10.1371/journal.pone.0111430 (PMC4232242; doi:10.1371/journal.pone.0111430)
Supplement: Table S6 — DNA content in CaCl2 transfected cells for luciferase (A) and qPCR (B) measurements. HEK293T cells were transfected with different amounts of plasmids to conduct qPCR and luciferase measurements (see results in figures 4 and 5). The indicated molar ratio of each plasmid was added to the transfection medium for each condition. (PDF) [file pone.0111430.s008.pdf]

A

	pcDNA3-Nkx2.2	Amaxa-GFP
Nkx2.2	3	1.5
control	0	4.5

B

	promoterless pGL4.12	pGL4.12-Irx3	pRL-TK	pcDNA3-Nkx2.2	Amaxa-GFP
Irx	0	0.5	0.1	0	3.5
pB	0.5	0	0.1	0	3.5
N1	0	0.5	0.1	0.5	3
N2	0	0.5	0.1	1	2.5
N3	0	0.5	0.1	1.5	2
pB_N	0.5	0	0.1	1.5	2

Table S-1 DNA content in $CaCl_2$ transfected cells for luciferase (A) and qPCR (B) measurements. HEK293T cells were transfected with different amounts of plasmids to conduct qPCR and luciferase measurements (see results in figures 4 and 5). The indicated molar ratio of each plasmid was added to the transfection medium for each condition.
